# TON1 recruiting motif 21 positively regulates the flavonoid metabolic pathway at the translational level in *Arabidopsis thaliana*

**DOI:** 10.1007/s00425-024-04337-x

**Published:** 2024-02-08

**Authors:** Ling Wu, Xuan Chen, Ping Zhang, Shaowei Yan, Tingzhi Zhang, Yuanyuan Li

**Affiliations:** 1 College of Life Sciences, Hunan Normal University, Changsha, 410081 Hunan Province China; 2 State Key Laboratory of Chemo/Biosensing and Chemometrics, College of Biology, and Hunan Key Laboratory of Plant Functional Genomics and Developmental Regulation, Hunan University, Changsha, 410082 Hunan Province China; 3 Syoung Cosmetics Manufacturing Co., Ltd., Changsha, 410000 Hunan Province China; 4 Changsha Yuelu Experimental High School, Changsha, 410000 Hunan Province China

**Keywords:** *Arabidopsis*, Flavonoid, Metabolomic, TON1 recruiting motif 21, Translation

## Abstract

**Main conclusion:**

This study reveals that *TRM21* acts as a positive regulator of flavonoid biosynthesis at the translational level in *Arabidopsis*, impacting both secondary metabolites and genes associated with root hair growth.

**Abstract:**

TRM (TONNEAU1-recruiting motif) superfamily proteins are reported to be involved in microtubule assembly. However, the functions of this protein family are just beginning to be uncovered. Here, we provide metabolomic and genetic evidence that 1 of the 34 *TRM* members, *TRM21*, positively regulates the biosynthesis of flavonoids at the translational level in *Arabidopsis thaliana*. A loss-of-function mutation in *TRM21* led to root hair growth defects and stunted plant growth, accompanied by significant alterations in secondary metabolites, particularly a marked reduction in flavonoid content. Interestingly, our study revealed that the transcription levels of genes involved in the flavonoid biosynthesis pathway remained unchanged in the *trm21* mutants, but there was a significant downregulation in the translation levels of certain genes [*flavanone 3-hydroxylase* (*F3H*), *dihydroflavonol-4-reductase* (*DFR*), *anthocyanidin reductase* (*ANR*), *flavanone 3’-hydroxylase* (*F3'H*), *flavonol synthase* (*FLS*), *chalcone synthase* (*CHS*)]. Additionally, the translation levels of some genes related to root hair growth [*RHO-related GTPases of plant 2* (*ROP2*), *root hair defective 6* (*RHD6*), *root hair defective 2* (*RHD2*)] were also reduced in the *trm21* mutants. Taken together, these results indicate that *TRM21* functions as a positive regulator of flavonoid biosynthesis at the translational level in *Arabidopsis*.

**Supplementary Information:**

The online version contains supplementary material available at 10.1007/s00425-024-04337-x.

## Introduction

Flavonoids are representative plant secondary metabolites with significant roles in regulating plant growth and responses to stress (Taylor and Grotewold 2005; Lepiniec et al. 2006; Daryanavard et al. 2023). The accumulation of flavonoids is influenced by various developmental signals, including plant hormones such as abscisic acid, jasmonate, brassinosteroids and auxin (Peng et al. 2011; Qi et al. 2011; Jiang et al. 2022; Shi et al. 2022), as well as factors such as sucrose (Teng et al. 2005; Solfanelli et al. 2006), temperature (Zhang et al. 2011), UV irradiation (Cominelli et al. 2008), drought (Nakabayashi et al. 2014a, b), and nitrogen (Liang and He 2018). Furthermore, they are also implicated in root hair development (Gayomba and Muday 2020; Struk et al. 2022) and play a role in cell wall restoration (Saffer and Irish 2018). The fine-tuned spatial and temporal regulation of flavonoid biosynthesis in *Arabidopsis* is achieved via a complex network of various transcription factors (Winkel-Shirley 2001; Koes et al. 2005; Lepiniec et al. 2006; Xu et al. 2015). The regulators *myeloblastosis 11* (*MYB11*), *myeloblastosis 12* (*MYB12*), and *myeloblastosis 111* (*MYB111*) regulate the expression of the four early flavonoid biosynthetic genes *chalcone synthase* (*CHS*), *chalcone isomerase* (*CHI*), *flavanone 3-hydroxylase* (*F3H*), and *flavonol synthase 1* (*FLS1*) (Stracke et al. 2007, 2010), whereas the anthocyanin regulators *myeloblastosis 75* (*MYB75*), *myeloblastosis 90* (*MYB90*), *myeloblastosis 113* (*MYB113*), and *myeloblastosis 114* (*MYB114*) control the transcription levels of *dihydroflavonol-4-reductase* (*DFR*) and *anthocyanidin synthase* (*ANS*) (Borevitz et al. 2000; Gonzalez et al. 2008; Dubos et al. 2010).

In addition to protecting plants against various biotic and abiotic stresses in their environment (Winkel-Shirley 2001), flavonoids play a crucial role in human health, such as by providing anticancer, anti-inflammatory, and cardiovascular preventive activities (Ross and Kasum 2002; Lee et al. 2007; Kozłowska and Szostak-Wegierek 2014). Owing to their significance in plant physiological processes and human nutrition, flavonoids have been one of the most intensively studied plant secondary metabolites over the last several decades.

Plant cells have no capacity to migrate, and their location is irreversibly established through oriented cell divisions occurring in specialized histogenic tissues called meristems (Spinner et al. 2013; Schaefer et al. 2017). Plant cells established their plane of division early during the cell cycle, and one of the first conspicuous signs of commitment to division is the transition of the cortical microtubular array into a dense preprophase band (PPB) encircling the nucleus (Spinner et al. 2013; Schaefer et al. 2017). PPB delineates the cortical division site and plays a crucial role in determining the division plane at the onset of mitosis (Mineyuki 1999). Only a few mutations specifically affect PPB formation. Tonneau 1 (TON1) (Traas et al. 1995; Azimzadeh et al. 2008) and FASS/Tonneau 2/Discordia 1 (FASS/TON2/DCD1) are two specific proteins directly involved in PPB formation (Spinner et al. 2013). The Tonneau 1- Tonneau1-recruiting motif protein-phosphatase 2A (TON1-TRM-PP2A, TTP) complex is a protein network that regulates the transition from interphase to the premitotic microtubule array (Drevensek et al. 2012; Spinner et al. 2013). Within the complex, the TRM superfamily plays a role in complex assembly and targeting to the cytoskeleton (Spinner et al. 2013; Schaefer et al. 2017).

A recent study revealed that *TRM7* expression is cell cycle-regulated and plays a critical role during specific stages of cell division; *trm678* mutant plants were smaller and lost growth capacity and development robustness (Schaefer et al. 2017). *TRM4* plays an important role in mucilage cellulose deposition, cellulosic ray length and the establishment of mucilage architecture (Yang et al. 2018) in *Arabidopsis*. *TRM21* is part of *TRM* Group 5, together with *TRM17*, *TRM19*, *TRM20*, and *TRM26* (Drevensek et al. 2012), and the biological functions of this group’s protein remain largely unclear.

In this work, we created two independent *trm21* single mutants (*trm21-1*, *trm21-2*) and a *trm21-3/trm20* double mutant line using CRISPR/Cas9 technology. We demonstrated that *trm21* mutants exhibited root hair growth defects and sensitivity to low nitrogen, abscisic acid (ABA) and salt stress. Nontargeted metabolite profiling using liquid chromatography‒mass spectrometry (LC‒MS) was performed to identify changes in secondary metabolites and intermediate concentrations in various pathways in the *trm21-1* mutants. Interestingly, our study revealed that *TRM21* is required for the mRNA translation of genes involved in flavonoid biosynthesis and root hair growth.

## Materials and methods

### Construction of plasmids

The chimeric sgRNA for *TRM21* was constructed by cloning the annealed oligos *TRM21*-sgF and *TRM21*-sgR into the M2CRISPR vector.

For generating *TRM21pro::TRM21/trm21* transgenic plant, a 1.4-kb fragment upstream of the ATG start codon of *TMR21* was amplified from *Arabidopsis* gene using primers TRM21pro-F and TRM21pro-R and cloned into the vector pHB at the EcoRI and HindIII restriction sites to produce the construct *TRM21pro-pHB* by homologous recombination. Then, the coding sequence of *TRM21* was amplified from Arabidopsis cDNA using primers TRM21-pHB-F and TRM21-pHB-R and cloned into the construct *TRM21pro-pHB* at the BamHI and XbaI restriction sites to produce the construct *TRM21pro::TRM21/trm21* by homologous recombination. The primers are listed in Suppl. Table S1.

### Plant materials and growth conditions

*Arabidopsis thaliana* Col-0 was used as the WT background in this study. The *trm21-1* and *trm21-2* mutants were generated by knocking out *TRM21* (*At5g43880*) from the WT background using CRISPR–Cas9. The double mutant *trm21-3/20* was generated by knocking out the *TRM21* and *TRM20* (*At4g28760*) genes from the WT background using CRISPR–Cas9. For generating *TRM21pro::TRM21/trm21* transgenic plant, the *trm21-1* mutant was transformed with vector *TRM21pro::TRM21-pHB* using the floral dip method (Clough and Bent 1998).

*Arabidopsis thaliana* seeds were washed with 75% (v/v) ethanol for 7 min and surface sterilized with bleach [3% (w/v) sodium hypochlorite] for 5 min in the clean bench, and rinsed four times with sterile water. The sterilized seeds were cold-stratified at 4 °C for 3 days and then sown on half-strength Murashige and Skoog (1/2 MS) medium containing 1% (w/v) agar and 0.8% (w/v) sucrose. The plates were placed in a growth chamber with a 16/8-h light/dark cycle at 22 °C.

### Phenotypic analysis

For root hair length measurements, seeds were sown onto 1/2 MS medium, after 4 days of growth, root hairs approximately 0.6–0.8 mm from the root tip were captured using an Olympus SZX16 stereomicroscope. Root hair length was then measured from the digital images using ImageJ software 1.52e.

For the N response assay, seeds were stratified for 3 days and then sown on 1/2 MS medium with different concentrations of nitrogen (Song et al. 2022). The seeds were germinated and grown on 1/2 MS medium with varying nitrogen concentrations for 6 days. The fresh weight and chlorophyll content of the plants were examined.

For the germination phenotypes under ABA treatment conditions, seeds were stratified at 4 °C for 3 d and grown on 1/2 MS medium with ABA at different concentrations for 6 days. At least 40 seeds for every line were used in one experiment, and at least 3 biological replicates were conducted.

For the NaCl treatment, seeds were surface-sterilized and germinated on 1/2 MS medium supplemented with 1% (w/v) sucrose and solidified with 0.9% (w/v) agar. NaCl was added into the medium at specified concentrations.

To measure root length, seeds were sown on 1/2 MS medium and allowed to germinate and grow for 3 days. Seedlings were transferred to 1/2 MS medium with ABA or NaCl at the indicated concentrations and grown for 6 days. At least 40 roots were assessed in all experiments, which were carried out with at least three biological replicates.

### Statistical analysis

SPSS Statistics 17.0 software (IBM, USA) was used for all statistical analyses. The data are shown as means ± standard deviation (SD). The significance was based on the *P*-values of < 0.05, < 0.01, and < 0.001, as determined by one-way ANOVA.

### Chlorophyll content analysis

The leaf chlorophyll content was measured as described previously (Song et al. 2022). Collect 40 fresh seedling leaves and ground with 1 mL of 80% acetone in a glass grinder (Fisher Scientific). Centrifuge the mixture at 13,000 g for 5 min at 4 °C. Measure the supernatant using a multimode microplate reader (PerkinElmer, EnSpire 2300) at wavelengths of 646.8 and 663.2 nm to estimate the total chlorophyll content.

### Liquid chromatography coupled with mass spectrometry (LC–MS) analysis

For mass spectrometry analysis, 1-month-old *Arabidopsis* seedlings were used. Plant tissues were ground into a fine powder in liquid nitrogen, and an appropriate amount of pre-cooled MeOH: ACN: H_2_O solution (volume ratio 2:2:1) was added. The mixture was subjected to 10 min of sonication. Wait at − 20 °C for 1 h, centrifugation at 13,000 g and 4 °C for 15 min, the supernatant was collected and freeze-dried. During mass spectrometry analysis, an appropriate amount of ACN: H_2_O solution (volume ratio 1:1) was added for reconstitution. The mixture was vortexed for 30 s and sonicated for 10 min. After another centrifugation at 13,000 g and 4 °C for 15 min, the supernatant was aspirated into the injection vial for LC–MS/MS analysis. In addition, 10 μL of each sample was mixed to prepare a quality control (QC) sample, which was also loaded into the injection bottle.

All chromatographic separations were performed using an ultra-performance liquid chromatography (UPLC) system (SCIEX, UK). ACQUITY UPLC T3 column (100 mm × 2.1 mm, 1.7 µm, Waters, UK) was used for the reversed phase separation. The column temperature was maintained at 50 °C, and the flow rate was set at 0.3 mL/min. The mobile phase consisted of solvent A (0.1% formic acid in water) and solvent B (0.1% formic acid in ACN). The gradient elution conditions were as follows: 0–0.5 min, 5% B; 0.5–2.5 min, 5% to 70% B; 2.5–7.5 min, 70–100% B; 7.5–9.0 min, 100%, 9.0–9.5 min, 100–5%; 9.5–12 min, 5% B.

Metabolite features from the samples were collected in both positive and negative ion modes by high-resolution mass spectrometry using Triple TOF 5600 +. The detailed parameters were as follows: Ion Source Gas1: 50 psi, Ion Source Gas2: 50 psi, Curtain Gas: 35 psi, Source Temperature: 500 °C, IonSapary Voltage Floating: 5500 V & − 4500 V (positive & negative); Declustering Potential (DP): ± 80 V (positive & negative); TOF MS scan m/z range: 60–1200 Da, Product ion scan m/z range: 25–1200 Da, TOF MS scan accumulation time 0.25 s/spectra, Product ion scan accumulation time 0.03 s/spectra; The secondary mass spectrometry was obtained using Information Dependent Acquisition (IDA) and was in High Sensitivity mode, CE: 30 V ± 15.

### Anthocyanins analysis

Anthocyanins analysis was performed essentially as described previously with some modifications (Liang and He 2018; Jiang et al. 2022). 7-day-old seedings were ground to fine powder in liquid nitrogen, 0.05 g of seedling tissue was extracted with 300 μL extraction buffer (methanol with 1% hydrochloric acid) overnight at 4 °C in darkness. Subsequently, 200 μL of water and 200 μL of chloroform were added, and the mixture was centrifuged at 14,000 g for 2 min to remove tissue debris. Supernatant solution was then measured at 530 nm. The content of anthocyanins in the wild type was set as value of 100%, and their levels in mutant and transgenic lines were compared with the wild type.

### RT‑qPCR analysis

Total RNA preparation, first-strand cDNA synthesis, and RT‑qPCR were performed as previously described (Jiang et al. 2022) with some modifications. For the *TRM21*, *TRM20* tissue-specific expression analysis, total RNA from root, stem, leaf, flower, and silique were isolated using TRIzol reagent (Ambion, 15596-026) and digested with DNase I (Takara, 2270A) to remove genomic DNA. First-strand cDNA was synthesized with a cDNA synthesis kit (Fermentas, K1622) according to the manufacturer’s instructions. RT‑qPCR analysis was performed on a CFX Connect real-time PCR detection system (Bio-Rad) using SYBR green reagent (Roche, 4913914001). For the transcript abundance analysis of several flavonoid pathway genes, 7-days-old seedlings were used for RNA isolated, as described above. ACTIN was used as a reference in RT‑qPCR analysis. Data are shown as mean expression ± SD. Gene-specific primers are listed in Suppl. Table S1.

### Polysome profiling and RT-qPCR assay

Polysome profiling assays were performed essentially as described previously (Missra and von Arnim 2014), with some modifications. Briefly, total proteins were extracted from 9-days-old WT, *trm21-1*,* trm21-2*, *TRM21pro::TRM21/trm21-1* and *TRM21-3/20* seedlings. Then, 800 µL of supernatant was loaded onto a 36 mL continuous sucrose gradient in a polycarbonate tube and spun in a Beckman SW 32 Ti rotor at 17,000 g at 4 °C for 5 h. Sucrose gradients (15–60%) were prepared by layering 15% (w/v) sucrose on top of an equal volume of 60% (w/v) sucrose in 38 mL polycarbonate centrifuge tubes (Beckman Coulter). Eleven fractions were collected by carefully pipetting samples from the top of the gradient. The polysomal and non-polysomal fractions were determined based on UV absorption profiles obtained from identical but separate experiments. The top seven fractions (1–7) contained ribosome-free mRNAs and monosomes, and the bottom four fractions (8–11) contained mRNAs associated with multiple ribosomes. The RNA from the bottom four fractions was extracted using RNAZol reagent (MRC) and reverse transcribed using random hexamers. RT‑qPCR was performed as above, with ACTIN transcripts used as a negative control. The primers are listed in Suppl. Table S1.

## Results

### Phenotype analysis of *trm21* mutants

**Fig. 1:**
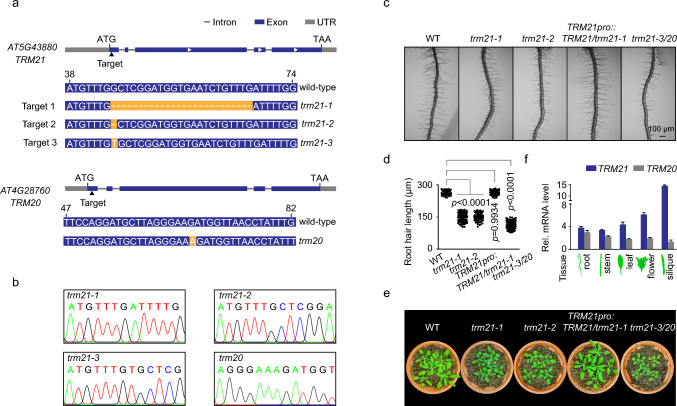
Phenotype analysis of *trm21* mutants. **a** Diagram of the CRISPR‒Cas9 mutant targets of the *TRM21* and *TRM20* genes. The mutation sites are highlighted in yellow, and the dashed line indicates missing bases. **b** Sequence peaks correspond to the mutated sites in the *TRM21* and *TRM20* genes. **c** Representative images of root hairs from 4-d-old WT (Col-0), *trm21-1*, *trm21-2*, *TRM21pro::TRM21/trm21-1*, and *trm21-3/trm20* seedlings. All images were acquired using an Olympus SZX16 stereomicroscope with the same settings. Scale bar, 100 μm. **d** Mean root hair length in the WT, *trm21-1*, *trm21-2*, *TRM21pro::TRM21/trm21-1*, and *trm21-3/trm20* lines. Root hair length was measured from the digital images using ImageJ software 1.52e. Approximately 80–160 root hairs from 8–10 roots per genotype were measured. The data are shown as the means ± standard deviations (SD); *P* < 0.001 for *trm21-1*, *trm21-2*, and *trm21-3/trm20*, *P* = 0.9934 for *TRM21pro::TRM21/trm21-1* as determined by one-way ANOVA relative to the WT. **e** Phenotype of 45-d-old WT, *trm21-1*, *trm21-2*, *TRM21pro::TRM21/trm21-1*, and *trm21-3/trm20* lines under normal soil culture conditions. **f** Analysis of *TRM21* and *TRM20* expression in different tissues, including roots, stems, leaves, flowers, and siliques. The data are shown as the means ± SD; ***P < 0.001, n.s., no significance, one-way ANOVA. All assays were performed in at least three biological replicates, yielding similar results

To determine the roles of *TRM21* in plant growth and environmental adaptation, we created transgenic plants with mutations in the *TRM21* gene by applying CRISPR/Cas9 technology. By performing PCR-based gene sequence analysis, we isolated two independent *trm21* single mutants (*trm21-1, trm21-2*) and a *trm21-3/trm20* double mutant (Fig. 1a, b). We found that *trm21-1* lost 26 base pairs at 45–66 bp downstream of the translational start site of *TRM21* compared to the wild type. Similarly, *trm21-2* lost the 45th base pair of *TRM21* compared to the wild type. For the *trm21-3/trm20* double mutant, a T-A base pair was inserted before the 45th base pair at the translational start site of *TRM21*, and an A-T base pair was inserted before the 66th base pair at the translational start site of *TRM20* (Fig. 1a, b). We transformed *trm21-1* with *TRM21* cDNA driven by the *TRM21* promoter and obtained the complementary *TRM21pro::TRM21/trm21-1* line.

We characterized the phenotypes of the two *trm21* lines, along with the *TRM21pro::TRM21/trm21-1* and *trm21-3/trm20* transgenic lines. Notably, the *trm21* lines, but not the *TRM21pro::TRM21/trm21-1* line, showed shorter root hairs than the WT, whereas the *trm21-3/trm20* double mutants showed severe root hair defects (Fig. 1c, d). Furthermore, *trm21-1, trm21-2* and *trm21-3/trm20* showed smaller rosettes than WT and *TRM21pro::TRM21/trm21-1* (Fig. 1e)*.* We investigated the expression patterns of *TRM21* and *TRM20* (Fig. S1). Transcripts of *TRM21* and *TRM20* were identified in 45-day-old seedlings of wild-type *Arabidopsis*, including roots, stems, leaves, flowers, and siliques (Fig. 1f). The expression of *TRM21* was significantly higher in siliques than in other organs, and the expression level of *TRM20* was lower in all tissues than that of *TRM21* (Fig. 1f).

### *TRM21* is required for plant responses to stress

**Fig. 2:**
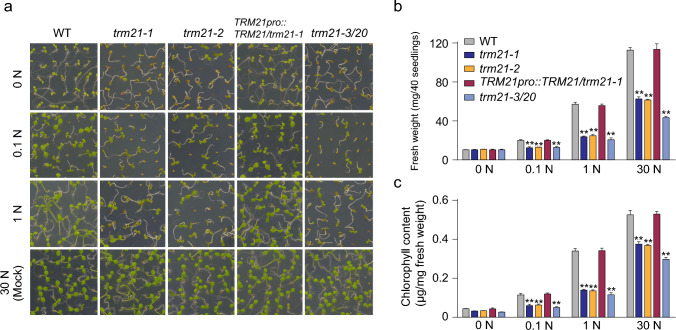
*TRM21* is involved in the N response. **a** Phenotypic analysis of WT, *trm21-1*, *trm21-2*, *TRM21pro::TRM21/trm21-1*, and *trm21-3/trm20* plants grown on 1/2-strength Murashige and Skoog medium containing varying concentrations of nitrogen (N) [N deficiency; 0, 0.1, 1, and 30 mM (as normal condition)] for 6 days. At least three independent experiments were performed, and similar results were obtained. **b, c** Fresh weight and chlorophyll content of distinct genotypes grown under different N concentrations, as shown in **a**. Forty seedlings were pooled for each measurement, and the values are the means ± SD of three biological replicates. The data are shown as the means ± SD; **P < 0.01, n.s., no significance, one-way ANOVA. All assays were performed in at least three biological replicates, yielding similar results

To determine whether *TRM21* was involved in responses to biotic and abiotic stress. N response assays were conducted to examine the performance of the WT, *trm21-1, trm21-2, TRM21pro::TRM21/trm21-1* and *trm21-3/trm20*. We performed this assay with early postgermination-stage plants on medium containing various N concentrations. Because 30 mM N is close to the N concentration of normal 1/2 MS medium, this concentration was regarded as the normal condition (mock) in this assay (Yasuda et al. 2014). All the seedlings exposed to N-deficient (0, 0.1, and 1 mM) conditions exhibited strongly reduced growth, especially the *trm21-1, trm21-2,* and *trm21-3/trm20* seedlings (Fig. 2a). Specifically, we detected a gradual increase in the fresh weight and chlorophyll content of seedlings as the N concentration increased (Fig. 2b, c). We found that *trm21-1, trm21-2,* and *trm21-3/trm20* exhibited enhanced sensitivity under N-deficient conditions compared with WT plants, although the fresh weight and chlorophyll content also gradually increased (Fig. 2). Our results indicated that *TRM21* is required for plant responses to low N nutrient levels.

Moreover, a germination rate assay on half-strength Murashige and Skoog (1/2 MS) medium alone or with the phytohormone ABA revealed that the *trm21* and *trm21-3/trm20* lines are more sensitive to ABA treatment than the WT or the complementary lines (Fig. S2). We thus exposed seedlings to high NaCl concentrations of 100 and 150 mM and established that root growth in the *trm21* and *trm21-3/trm20* lines was more inhibited than that in the WT (Fig. S3).

### Metabolomic analysis of the *trm21* mutant

Previous studies have reported the significant roles of secondary metabolites in the interactions between plants and their environment (Bennett and Wallsgrove 1994; Yang et al. 2018). These metabolites are involved in plant hormone signaling and protect plants from various biotic and abiotic stresses (Harbrne and Williams 2000; Winkel-Shirley 2001; Lepiniec et al. 2006). In particular, flavonoid compounds such as anthocyanins have been shown to enhance plant tolerance to low temperature, drought, and high salinity stress and improve plant resistance to low-nitrogen conditions (Liang and He 2018). Therefore, we sought to investigate whether *TRM21* influences the production of secondary metabolites within plants. To do so, we utilized liquid chromatography coupled with mass spectrometry (LC‒MS) to analyze the total metabolite content of 1-month-old WT and *trm21-1* mutant plant samples.

**Fig. 3:**
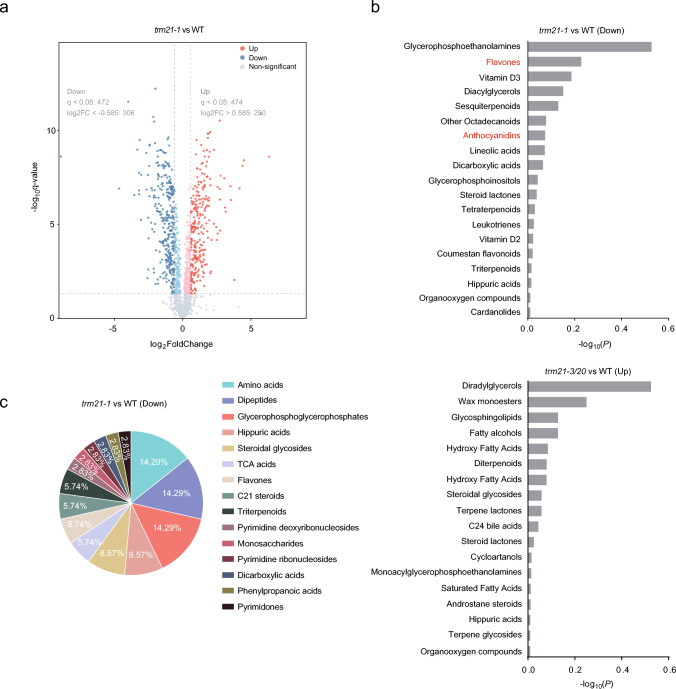
Metabolomic analysis of the *trm21* mutant. **a** Volcano plot showing the relative levels of upregulated and downregulated metabolites in *trm21-1* compared to the WT. **b** Gene Ontology term enrichment analysis of upregulated and downregulated metabolites in *trm21-1*. **c** Pie chart showing the enrichment of different types of downregulated metabolites in the *trm21-1* mutant

Principal component analysis (PCA) showed that the contribution ratios of principal components PC1 and PC2 were 57.5% and 30.6%, respectively, and the results showed identical repeatability of the same experimental group (Fig. S4a). In addition, the heatmap of metabolite levels revealed differences in metabolite profiles between the WT and *trm21* mutant (Fig. S4b). We identified 336 decreased metabolites and 291 increased metabolites by more than 0.585-fold in *trm21-1* relative to the WT (q < 0.05) (Fig. 3a, Table S2). GO analysis showed an enrichment of secondary metabolites such as flavones, anthocyanidins, and vitamin D among the decreased metabolites, while lipid-related metabolites were enriched among the increased metabolites (Fig. 3b). Further analysis of the decreased metabolites revealed that the most abundant were amino acids, dipeptides, and glycerophosphates, accounting for 42.87% of all metabolites. Following them were hippuric acids and steroidal glycosides, accounting for 17.14%. TCA acids, flavones, C21 steroids, and triterpenoids accounted for 5.74% each, while the other types of metabolites accounted for 2.83% (Fig. 3c). Taken together, these results indicate that *TMR21* is involved in regulating the production of secondary metabolites within plants.

### Loss-of-function mutation of* TRM21* affects both flavonoid and anthocyanidin accumulation but not the transcription of genes related to flavonoid biosynthesis in *Arabidopsis*

**Fig. 4:**
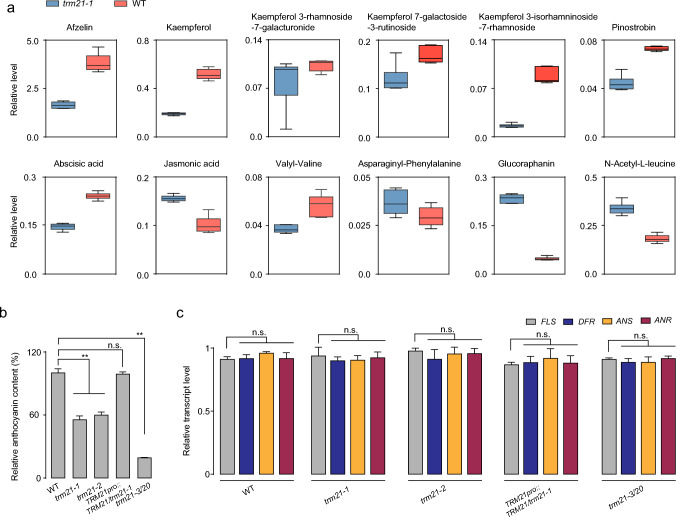
Loss-of-function mutation of *TRM21* affects both flavonoid and anthocyanidin accumulation but not the transcription of genes related to flavonoid biosynthesis in *Arabidopsis*. **a** Examples of compounds related to flavonoids, hormones, and amino acid metabolism that are affected by the *trm21* mutation under normal growth. **b** Relative anthocyanin levels in 7-d-old WT, *trm21-1*, *trm21-2*, *TRM21pro::TRM21/trm21-1*, and *trm21-3/trm20* seedlings. **c** RT‒qPCR assay showing the relative transcript abundance of genes involved in the flavonoid pathway. The data are shown as the means ± SD; **P < 0.01, n.s., no significance, one-way ANOVA. All assays were performed in at least three biological replicates, yielding similar results

We noticed that the expression levels of metabolites associated with flavonoids, hormones, and amino acid metabolism changed in *trm21-1* (Fig. 4a). Flavonols are the major flavonoid compounds present in *Arabidopsis* seedlings, particularly the three major flavonol glycosides Kaempferol 3-rhamnoside-7-galacturonide (K3R7G), Kaempferol 7-galactoside 3-rutinoside (K7G3R), and Kaempferol 3-isorhamninoside-7-rhamnoside (K3R7R) (Yonekura-Sakakibara et al. 2008; Stracke et al. 2009). HPLC analysis revealed that these three compounds were significantly decreased in the *trm21* knockout lines compared to WT seedlings (Fig. 4a).

Due to the crucial roles of flavonoid compounds in plants, we next investigated the mechanisms by which *TRM21* regulates flavonoid biosynthesis. We determined the anthocyanin content in two lines of *trm21* mutants, *trm21-3/20*, and complementation lines of *trm21* plants. A marked reduction in anthocyanin content was found in the seedlings of the *trm21* knockout lines in comparison to the WT (Fig. 4b), implying that anthocyanin was severely suppressed by *TRM21* in the transgenic *Arabidopsis*.

To investigate whether the transcript abundances of flavonoid pathway genes were affected by the loss-of-function mutation of *TRM21*, we used RT‒qPCR to measure the relative transcript abundance of several flavonoid pathway genes (Fig. 4c). The relative transcript levels of three key biosynthetic genes, *FLS*, *DFR*, *ANS* and *ANR*, remained unchanged in the knockdown seedlings (Fig. 4c). Moreover, RT‒qPCR analysis showed that several biosynthetic genes (e.g., *C4H*, *CHS*, *CHI*, *F3H*, and *F3’H*) and regulatory genes (e.g., *MYB11*, *MYB12*, *MYB111*, *MYB113*, *MYB114*) also remained unchanged in the knockdown seedlings (Fig. S5).

### *TRM21* is required for the mRNA translation in *Arabidopsis*

**Fig. 5:**
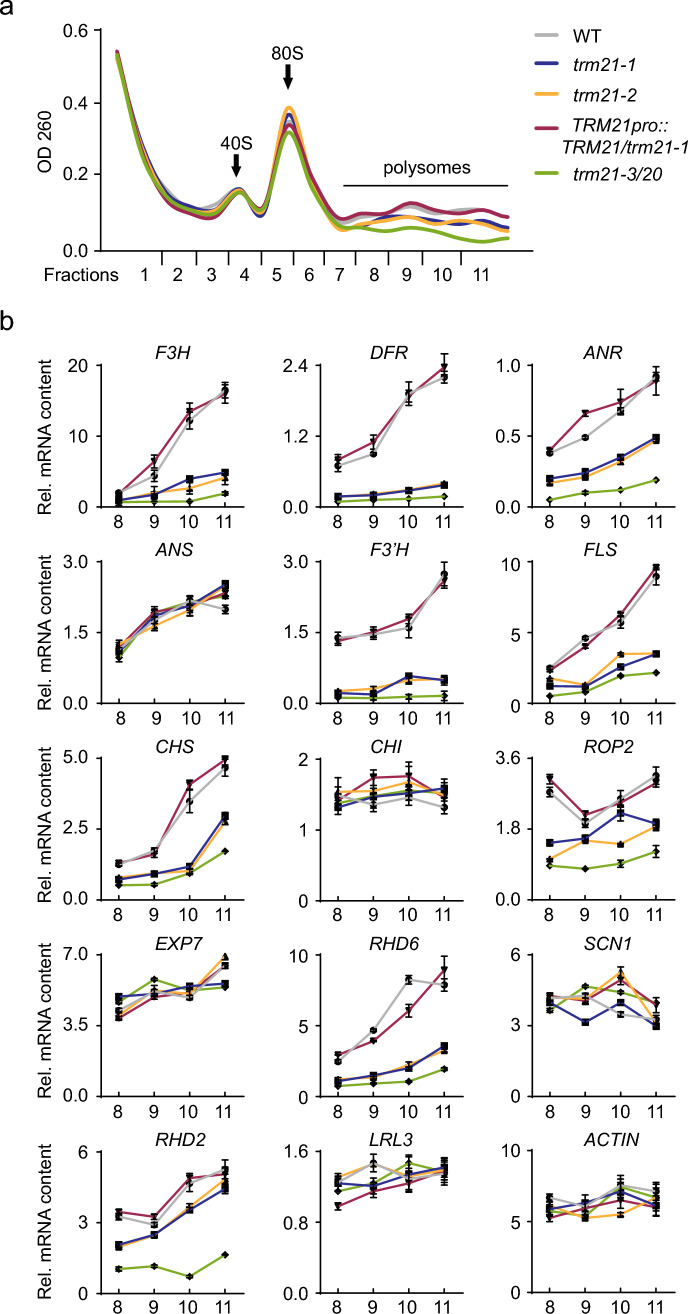
*TRM21* loss-of-function alters the translation of genes related to flavonoid biosynthesis in *Arabidopsis*. **a** Polysome profile assay showing protein synthesis in WT, *trm21-1*, *trm21-2*, *TRM21pro::TRM21/trm21-1*, and *trm21-3/trm20*. Fractions (8–11) containing mRNAs associated with polysomes are indicated with a black line. **b** RT‒qPCR assay showing polysome-associated mRNAs. All assays were performed in at least three biological replicates, yielding similar results

To further investigate the mechanism of *TRM21* in the plant development, we then tested whether *TRM21* is required for mRNA translation. Polysome profiling analysis was used to assess the effect of *TRM21* on the translation of flavonoid biosynthesis pathway genes and root hair growth genes. We noticed a decrease in the intensity of polysome fractions from polysome gradients on *trm21* mutants relative to the WT (Fig. 5a). Furthermore, RT‒qPCR assay analysis of genes with specific roles in the flavonoid compound biosynthetic pathways and root hair growth using polysome-associated mRNAs showed that the abundance of several genes (e.g., *F3H*, *DFR*, *ANR*, *F3’H*, *FLS*, *CHS*, *ANS*, *CHI*, *RHD27*) (which acts as a proxy for their translation levels) but not others (e.g., *ROP2*, *RHD6*, *EXP7*, *SCN1*, *LRL*3) in *trm21* mutants was lower. These results showed that *TRM21* is required for the mRNA translation of genes involved in flavonoid biosynthesis and root hair growth. However, the internal relationship between *TRM21* and the flavonoid pathway is still unknown.

## Discussion

The TRM superfamily complex has been identified as a group of proteins that interact with the TON1 protein (Drevensek et al. 2012), and recent studies have confirmed their critical roles in microtubule assembly (Schaefer et al. 2017; Yang et al. 2022). There are still many unknown functions of this protein complex. This study reveals that *TRM21* positively regulates the plant development and response to biotic and abiotic stresses. Specifically, *TRM21* modulates the translation levels of genes involved in the flavonoid biosynthesis pathway, rather than their transcription levels, to regulates the accumulation of flavonoids.

Plants employ many diverse metabolic pathways to produce > 200,000 compounds (Dixon and Strack 2003; Yonekura-Sakakibara et al. 2008). In addition to basic nutrients such as proteins, fats or carbohydrates, plants can produce other compounds, including taxoids, polysaccharides, flavones, etc. Secondary metabolites (SMs) are dispensable for plant metabolism and growth, whereas the wide variety and high diversity of secondary products are key components for plant growth and development, innate immunity, defense response signaling, and response to environmental stresses (Bennett and Wallsgrove 1994; Wink 2008; Yang et al. 2018). A recent study revealed that the amino acid glutamate plays a crucial role in activating long-distance, calcium-based defense signal transduction in plants (Kosmacz et al. 2020); flavonoids confer plant tolerance to low-nitrogen stress in *Arabidopsis* (Liang and He 2018); lipids serve as the primary constituents of cellular membranes, and plants can release fatty acids, which function as signaling compounds in response to environmental cues. In the *trm21-1* mutant, we observed decreased metabolites mostly associated with amino acids, carbohydrates, and the TCA cycle, while increased metabolites primarily involved lipid metabolism. This provides valuable clues for further investigating the biological functions of *TRM21* in the future.

The synthesis of flavonoids is finely regulated in both temporal and spatial dimensions. Recent research has revealed that *auxin response factor 2* (*ARF2*) regulates the accumulation of flavonoid compounds in plants by modulating the transcription levels of genes involved in the flavonoid biosynthesis pathway (Jiang et al. 2022). Anthocyanins contribute substantially to the low N tolerance of *Arabidopsis thaliana* (Liang and He 2018). We found that flavonoid compounds were significantly decreased in the *trm21* knockout lines compared to WT seedlings and *trm21* mutants exhibited enhanced sensitivity under N-deficient conditions. It suggests *TRM21* affects the flavonoid content to regulate plant response to N-deficient. We found that *TRM21* does not affect the transcription levels of key enzymes in the flavonoid biosynthesis pathway but instead regulates their translation to influence the accumulation of plant secondary metabolites. These findings uncovered the correlation between *TRM21* and flavonoid content in *Arabidopsis*.

Amino acids not only serve as the fundamental building blocks for protein synthesis but also play crucial roles as signaling molecules. In mammals and yeast, amino acids act as essential upstream signals for the *target of rapamycin* (*TOR*) kinase signaling pathway, a central regulator of cell proliferation and growth that integrates nutrient and hormone signaling (Fu et al. 2020; Liu and Sabatini 2020). Recent research in plants has shown that amino acids function as activating factors, influencing the reorganization of the actin cytoskeleton (Cao et al. 2019). Moreover, under conditions of inorganic nitrogen starvation, a study revealed that 15 proteinogenic amino acids act as signaling molecules to reactivate translation (Liu et al. 2021a, b). These findings highlight the crucial role of amino acids as signaling molecules in regulating normal plant cell growth, although the precise molecular mechanisms require further investigation. In our research, we observed significant downregulation of amino acid metabolism in the *trm21* mutant, which could potentially disrupt normal mRNA translation processes, leading to plant growth defects.

### Supplementary Information

Below is the link to the electronic supplementary material.

Supplementary file1 (PDF 3747 KB)

Supplementary file2 (XLSX 14 KB)

Supplementary file3 (XLSX 87 KB)

## Data Availability

Data supported the findings of this study are available in the Suppl. Tables S1-2.
