# Supplementary material for: TON1 recruiting motif 21 positively regulates the flavonoid metabolic pathway at the translational level in Arabidopsis thaliana
Source: Planta. 2024 Feb 8;259(3):65. doi: 10.1007/s00425-024-04337-x (PMC10853083; doi:10.1007/s00425-024-04337-x)
Supplement: Supplementary file 1 — Supplementary file1 (PDF 3747 KB) [file 425_2024_4337_MOESM1_ESM.pdf]

Suppl. Metarial

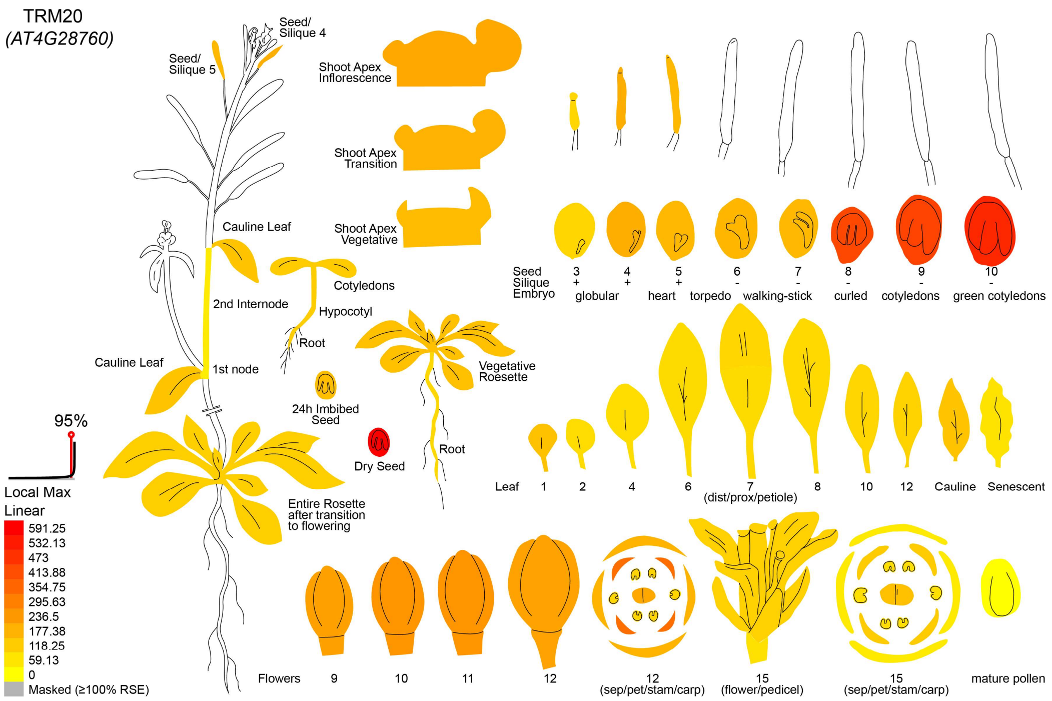

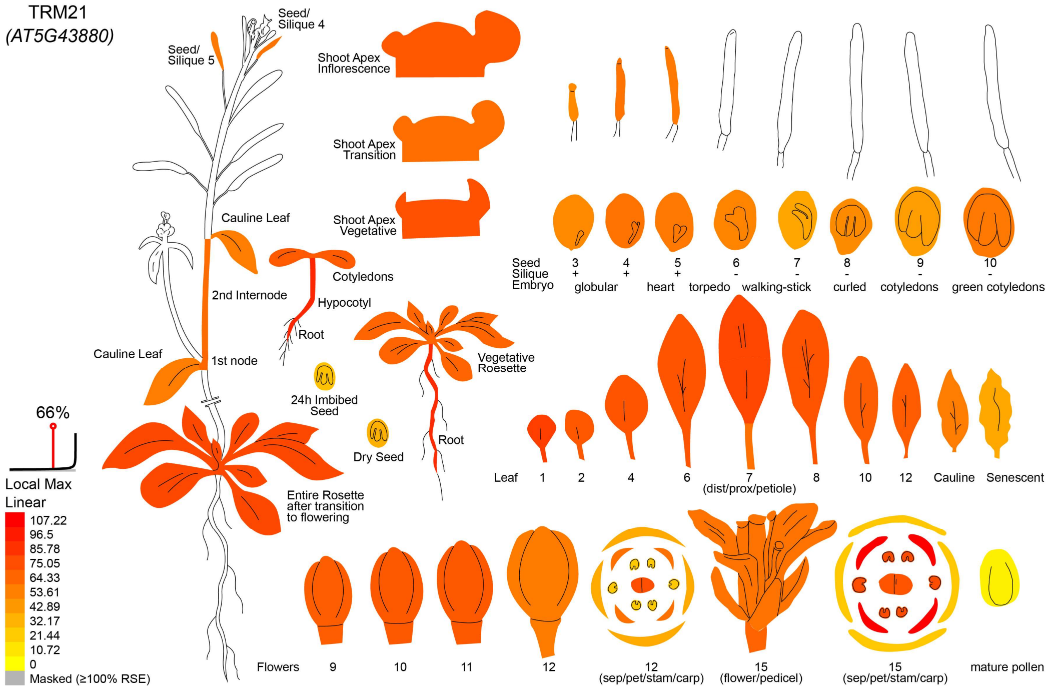

Fig. S1 *TRM20* and *TRM21* expression patterns. Gene expression levels were analyzed in ePlant (http://bar.utoronto.ca/eplant/)

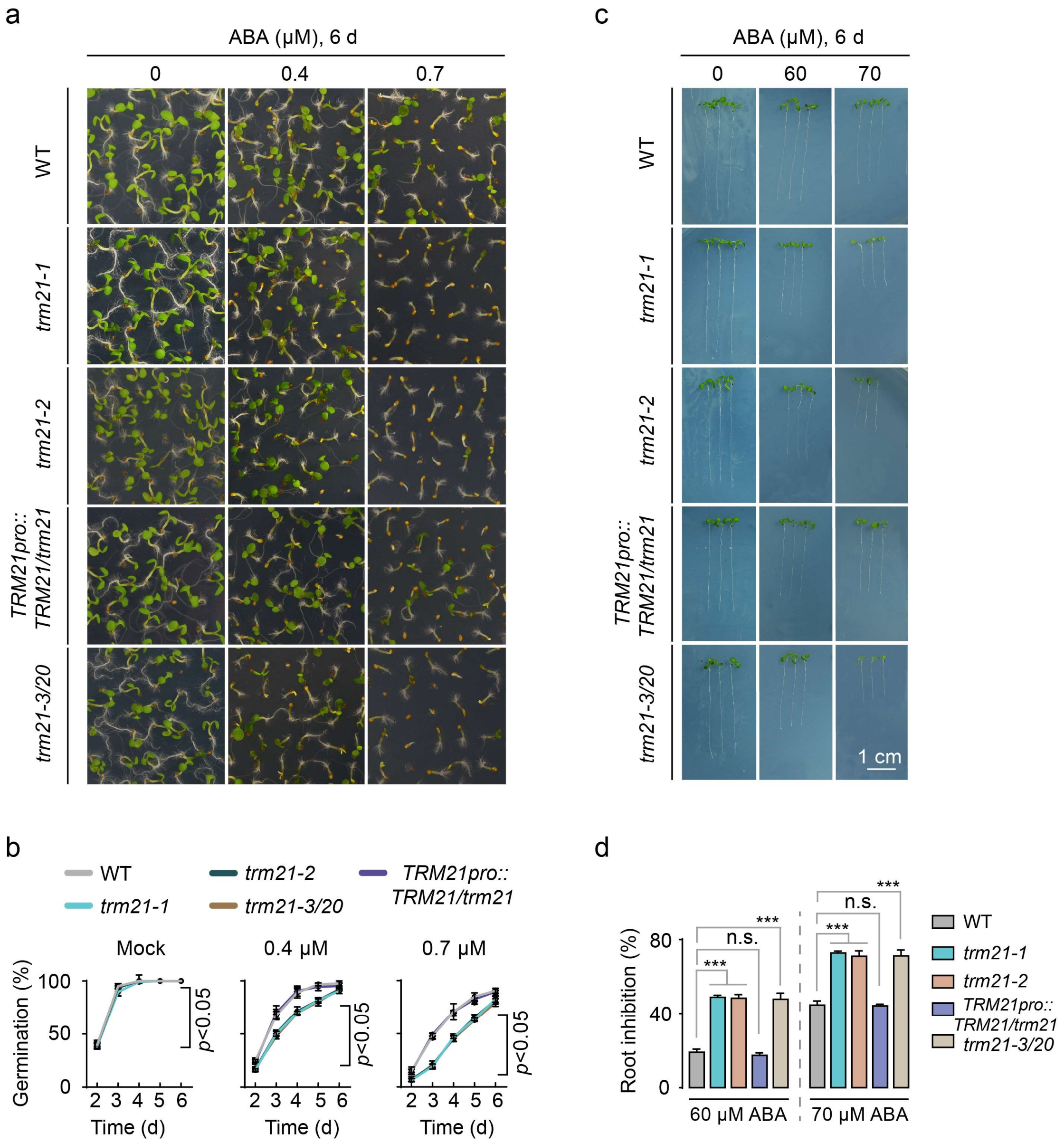

Fig. S2 *TRM21* functions in ABA response. **a** Representative images of seed germination from WT, *trm21-1*, *trm21-2*, *TRM21pro:: TRM21/trm21-1*, and *trm21-3/trm20* seeds sown on half-strength MS medium with 0.4 or 0.7 μM ABA. Images of 6-d-old seedlings are shown. **b** Germination rate over time of WT, *trm21-1*, *trm21-2*, *TRM21pro:: TRM21/trm21-1*, and *trm21-3/trm20* seedlings. Approximately 30–40 seeds of each line were assessed. **c** Representative images of 9-d-old WT, *trm21-1*, *trm21-2*, *TRM21pro:: TRM21/trm21-1*, and *trm21-3/trm20* seedlings. 3-d-old seedlings were transferred to half-strength MS medium with 0, 60, or 70 μM ABA and grown for 6 d. Scale bar, 1 cm. **d** Percentage of root inhibition following ABA treatment among the seedlings shown in **c**, relative to the no ABA control. At least 30 seeds of each line

were tested. The data are shown as means ± SD; ***$P < 0.001$, n.s., no significance, as determined by one-way ANOVA. All assays were performed in at least three biological replicates, yielding similar results

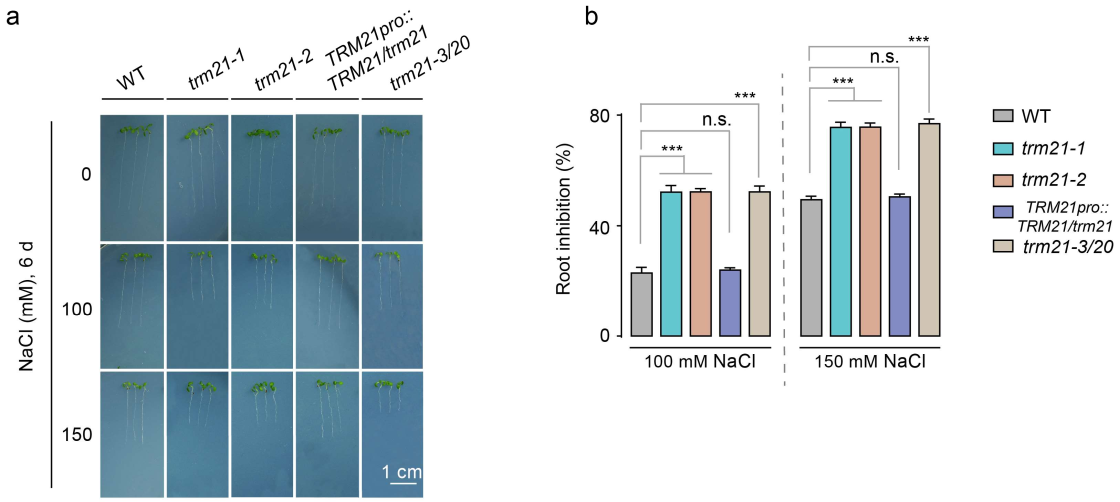

Fig. S3 *TRM21* functions in the salt stress response. **a** The *trm21* lines are more sensitive to salt stress than the WT. 3-d-old seedlings were transferred to half-strength MS medium containing NaCl and grown for 6 d on salt before taking the photographs. Three independent experiments were conducted for each treatment, showing the same results, and a representative picture is shown. **b** Root inhibition of WT, *trm21-1*, *trm21-2*, *TRM21pro:: TRM21/trm21-1*, and *trm21-3/trm20* lines under NaCl in **a**. At least 30 roots of each line were tested. The data are shown as the means ± SD; ***$P < 0.001$, n.s., no significance, as determined by one-way ANOVA

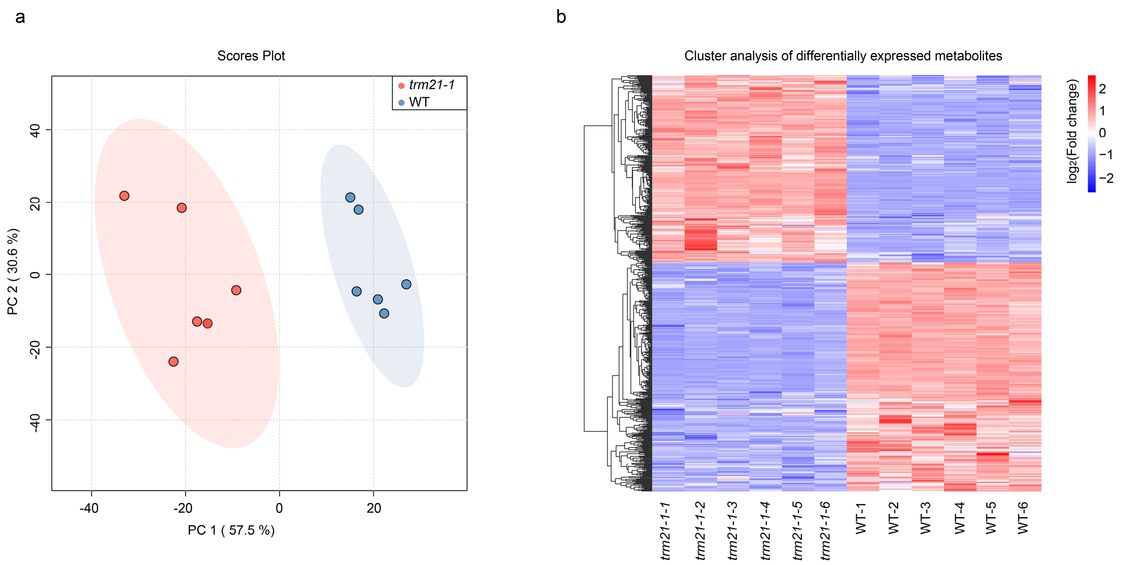

Fig. S4 Metabolomic analysis of the *trm21* mutant. **a** Principal component analysis plot of metabolites of one-month-old WT and *trm21-1* mutant plants. PC1 represents the difference between WT and *trm21-1*, while PC2 represents the variation within WT and *trm21-1*. **b** Clustering heatmaps of the relative levels of metabolites in the *trm21-1* mutant. Colors represent log_2-transformed fold changes in metabolite levels in the mutants relative to WT

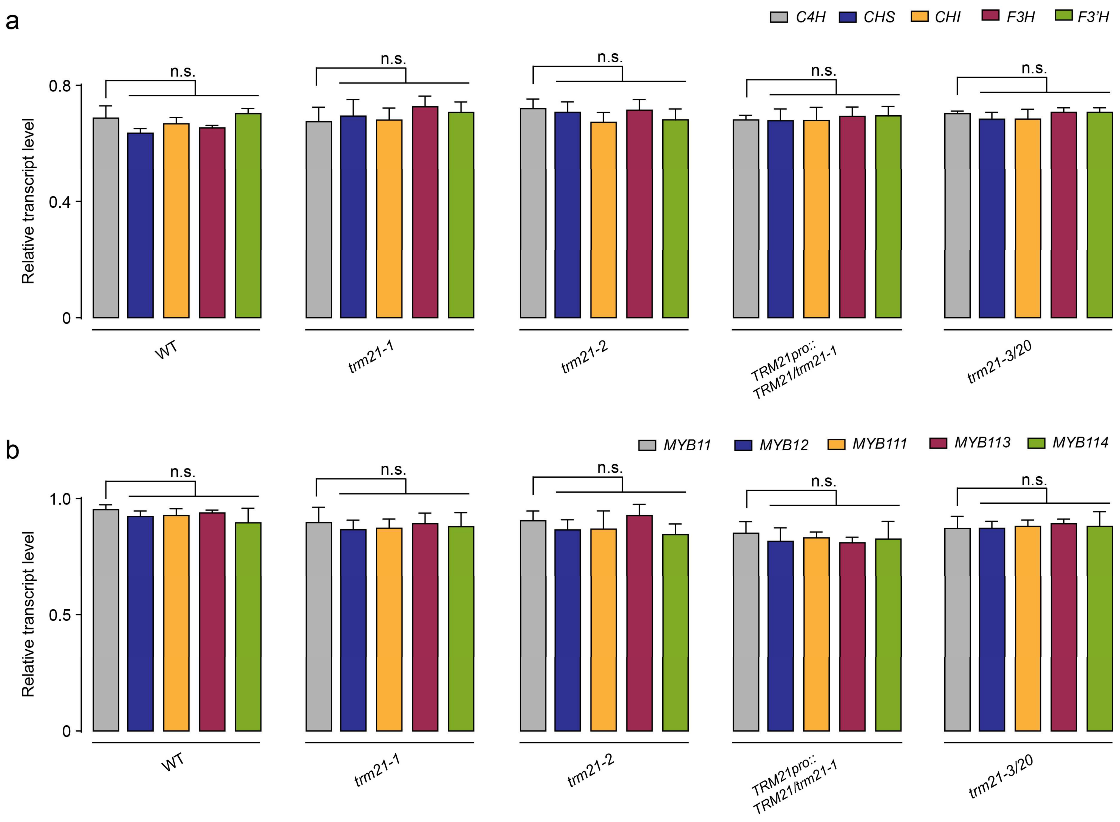

Fig. S5 Relative transcript abundance of the flavonoid pathway genes. **a** RT–qPCR analysis of the relative transcript abundance of genes involved in the flavonoid biosynthesis pathway. **b** RT–qPCR analysis of the relative transcript abundance of genes involved in regulating the flavonoid biosynthesis pathway. The data are shown as means ± SD; n.s., no significance, as determined by one-way ANOVA. All assays were performed in at least three biological replicates, yielding similar results
